# Supplementary figures and images for: Population structure, antimicrobial resistance, and virulence factors of diabetic foot-associated Escherichia coli
Source: Microbiol Spectr. 2026 Jan 13;14(2):e02837-25. doi: 10.1128/spectrum.02837-25 (PMC12889029; doi:10.1128/spectrum.02837-25)

Supplementary Figure 1

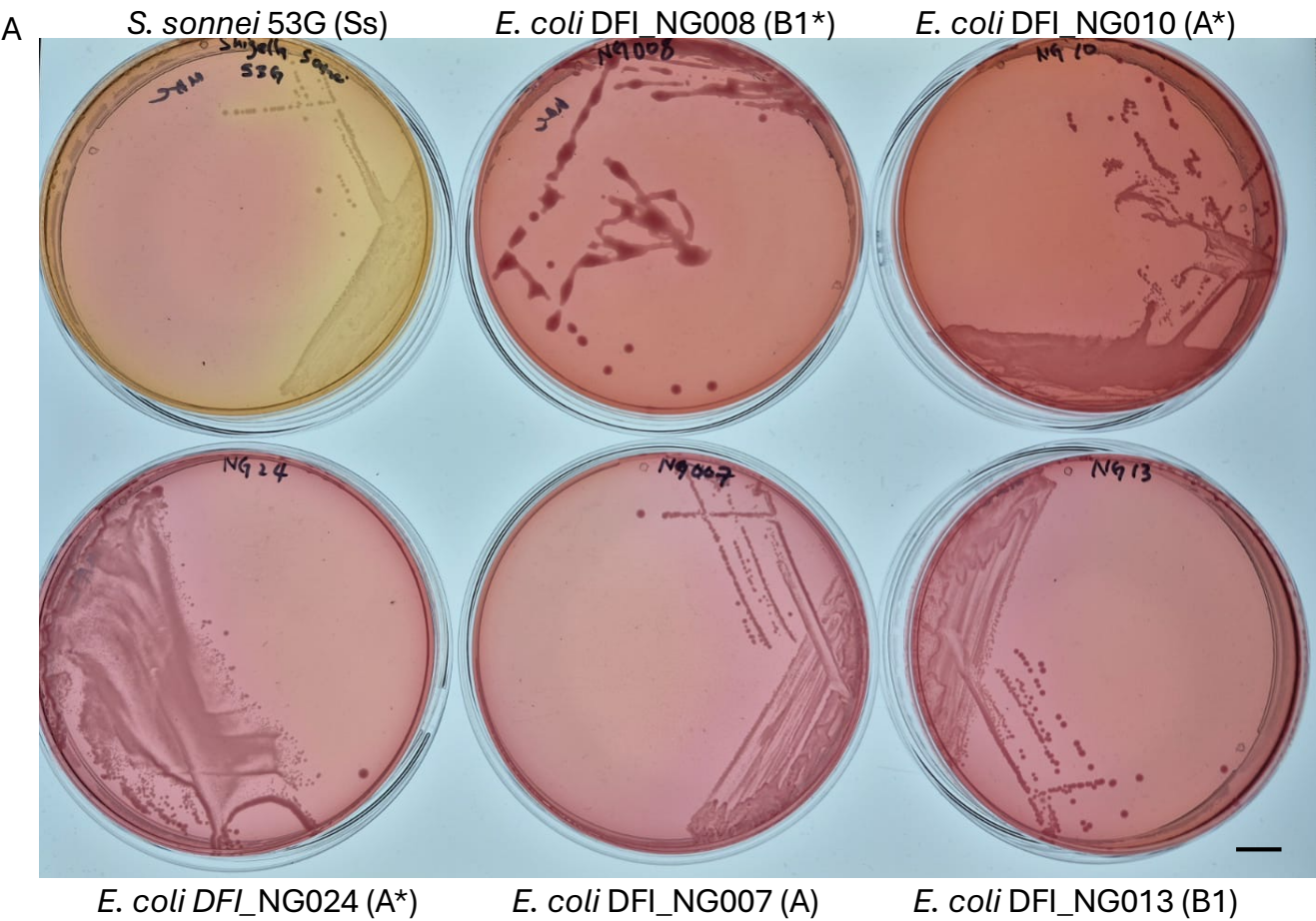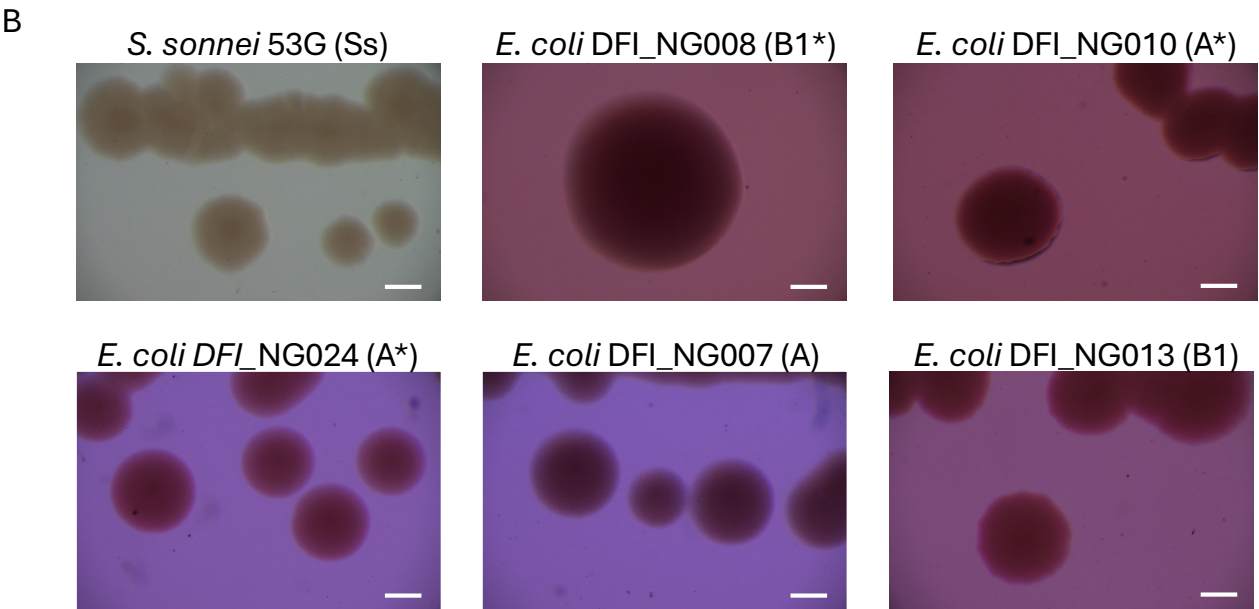

Supplement: Fig. S1 — Growth of representative DFEC strains on MacConkey agar. [file spectrum.02837-25-s0001.pdf]
