# Supplementary material for: Population structure, antimicrobial resistance, and virulence factors of diabetic foot-associated Escherichia coli
Source: Microbiol Spectr. 2026 Jan 13;14(2):e02837-25. doi: 10.1128/spectrum.02837-25 (PMC12889029; doi:10.1128/spectrum.02837-25)
Supplement: Supplemental material — Supplemental figure and table legends. [file spectrum.02837-25-s0002.docx]

**Supplementary Figure legends**

**Supplementary Figure 1. Growth of representative DFEC strains on MacConkey agar.** *S. sonnei* 53G, and the DFEC strains DFI_NG08, DFI_NG10. DFI_NG024, DFI_NG007, and DFI_NG013 were grown on MacConkey agar to test for the ability to ferment lactose. While *S. sonnei* 53G was unable to ferment lactose as expected, all the tested DFEC strains were able to ferment lactose, including the *Shigella*-like strains of A* and B1* lineages. Scalebars: (A) 1.0 cm; (B) 0.5 mm.

**Supplementary Table legends**

**Supplementary Table S1. Detailed summary of the characteristics of the Diabetic Foot-associated *E. coli* (DFEC) strains used in this study.** Different columns report strain name, accession, country of collection, phylogroup, O serotype, H serotype, K serotype, Enterobase and Pasteur MLST types, genome size, %GC content, sequencing approach, sequencing coverage, number of coding sequences (CDS), number of tRNAs, number of rRNAs, number of contigs and N50.

**Supplementary Table S2. List of gene presence/absence** **in DFEC strains**. Genomes were annotated using Prokka and the pangenome was reconstructed using Roary.

**Supplementary Table S3. % Occurrence of AMR phenotypes in DFEC strains per phylogroup.** AMR phenotypes were predicted using ABRicate.

**Supplementary Table S4. List of AMR marker presence/absence in DFEC strains.** AMR phenotypes were predicted using ABRicate.

**Supplementary Table S5. % Occurrence of ExPEC-related virulence factor in DFEC strains per phylogroup.** Virulence factors were predicted using ABRicate.

**Supplementary Table S6. List of virulence factor presence/absence in DFEC strains.** Virulence factors were predicted using ABRicate.

**Supplementary Table S7. List of metabolic reaction presence/absence in DFEC strains.** Metabolic reactions were predicted using CarveMe.
